# Culturally Adapted Problem-Solving Intervention for Women Experiencing Suicidal Ideation During Postnatal Period

**DOI:** 10.1192/bjo.2024.121

**Published:** 2024-08-01

**Authors:** Rabia Sattar, Nusrat Husain, Tayyeba Kiran, Ozlem Eylem, Nasim Chaudhry

**Affiliations:** 1 Pakistan Institute of Living and Learning, Lahore, Pakistan; 2 University of Manchester, Manchester, United Kingdom; 3 Mersey Care NHS Foundation Trust, Manchester, United Kingdom; 4 Pakistan Institute of Living and Learning, Rawalpindi, Pakistan; 5 Pakistan Institute of Living and Learning, Karachi, Pakistan

## Abstract

**Aims:**

Suicide is a global public health concern, affecting not only the individuals but also families. It is the leading cause of maternal death during pregnancy and up to one year after birth and commonly occurs after a period of suicidal ideation (SI). It is imperative to have interventions to help with SI and behaviors. We therefore aimed to adapt and test the feasibility and acceptability of a culturally adapted intervention for SI in women during postnatal period in Pakistan.

**Methods:**

This is a two phase, mixed method Randomized Controlled Trial (RCT). First phase included adaptation of an existing Culturally Adapted Manual-Assisted Problem-Solving intervention (CMAP) for women experiencing SI. Adaptation process included two focus group discussion (FGDs), one with lived experience experts (women who experienced suicidal ideation during postnatal period), the other with health professionals (n = 8 in each group). Second phase involves a feasibility RCT with aim to recruit and randomize a total of 90 postnatal women experiencing suicidal ideation (screened using the Beck Scale for Suicidal Ideation), randomize into either of two study arms: CMAP (n = 45) or Treatment as usual (n = 45). Potential participants are being recruited from hospitals, communities, and self-referrals from 5 major cities in Pakistan. Culturally adapted CMAP is a brief problem-solving therapy of 6 individually delivered sessions, lasting about 50 minutes. The primary outcome is to assess the feasibility of CMAP through semi-structured qualitative interviews. Secondary outcomes include measuring SI, self-harm, depression, social support, and quality of life. Assessments will be conducted at baseline and 3rd month post randomization.

**Results:**

Analysis of qualitative data from FGD with lived experience experts highlighted importance of incorporating additional techniques of trust building, modifying thinking behavior, mindfulness, distraction exercises including religious practices as a preventive measure of self-harm, child safety measures, and involvement of partner in intervention. Analysis of FGD with healthcare professionals emphasized addition of visualized content, re-assessing depression and suicidal ideation in-between the sessions to monitor relapse, involving family, and capacity building of health professionals to improve their understanding about perinatal mental health problems.

**Conclusion:**

Women in postnatal period are at high risk of SI, specifically those women from low- and middle-income countries, due to limited resources and mental healthcare provision. The earlier detection of SI, early intervention for suicide risk by delivery of culturally sensitive interventions can help reduce maternal mortality rates.

